# Endogenous Murine Leukemia Viruses: Relationship to XMRV and Related Sequences Detected in Human DNA Samples

**DOI:** 10.1155/2011/940210

**Published:** 2011-10-24

**Authors:** Oya Cingöz, John M. Coffin

**Affiliations:** Department of Molecular Biology and Microbiology and Genetics Program, Sackler School of Graduate Biomedical Sciences, Tufts University, Boston, MA 02111, USA

## Abstract

Xenotropic-murine-leukemia-virus-related virus (XMRV) was the first gammaretrovirus to be reported in humans. The sequence similarity between XMRV and murine leukemia viruses (MLVs) was consistent with an origin of XMRV from one or more MLVs present as endogenous proviruses in mouse genomes. Here, we review the relationship of the human and mouse virus isolates and discuss the potential complications associated with the detection of MLV-like sequences from clinical samples.

## 1. Endogenous MLVs

Retroviruses are unique in their requirement, as a natural step in their replication cycle, for integration into the genomes of the host cells they infect [1]. Through infection of the germ line, exogenous retroviruses can become a part of the host genome, leading to the generation of endogenous proviruses. All vertebrate species examined carry remnants of such prior retroviral infections in their genomes [1, 2]. Humans, for example, carry some 80,000 sequences or about 8% of our total genome, derived from retrovirus infections dating from some 40 million to a hundred thousand or so years ago. Mouse genomes contain a large number of more recently integrated endogenous proviruses; one of the best-studied groups comprises the murine leukemia viruses (MLVs). MLVs are thought to have entered the *Mus* germ line less than 1.5 million years ago [3, 4], and generation of new endogenous proviruses continues to this day. They can be classified based on their host range and sequence into ecotropic, xenotropic, polytropic and modified polytropic, MLVs [1, 5]. The host range of MLVs is determined by the species distribution of the receptors they use for entry. Ecotropic MLVs (of which there are only a few) use mCAT1, an allele of the cationic amino acid transporter found only in mice and a few other rodents [6]. The more common nonecotropic MLVs, including xenotropic (X-MLV), polytropic (P-MLV), and modified polytropic (MP-MLV) viruses use distinct alleles of Xpr1, a cell surface protein of unknown function, with X-MLVs unable to recognize the allele found in most inbred and a few wild mice, hence the name “xenotropic” (reviewed in [7]). The proviruses that correspond to these different viruses are referred to as *Xmv*, *Pmv*, and *Mpmv*, respectively. Experimentally, they are distinguished by hybridization to oligonucleotide probes specific for a sequence in the SU region of *env*. P-MLVs can be distinguished from MP-MLVs by a 27 bp insertion in their *env* genes [1, 5]. Endogenous nonecotropic proviruses are highly polymorphic in their genome location: inbred mice contain about 20 of each type and, on average, share about 30 with any other inbred strain. Of the 150 or so identified proviruses, only a few *Xmvs*, including* Bxv1* (*Xmv43*), are known to encode infectious virus [8]. No complete, replicating P-MLV or MP-MLV has ever been identified, although their *env* genes are often found as recombinants [1, 9].

**Figure 1:**
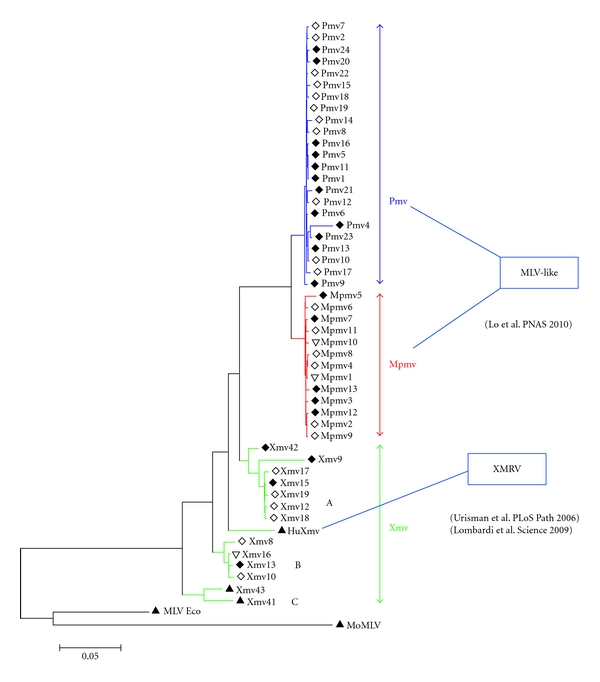
Relationship of proviruses in the sequenced mouse genome. The three groups of nonecotropic MLVs are indicated. Modified from Jern et al. [10].

**Figure 2:**
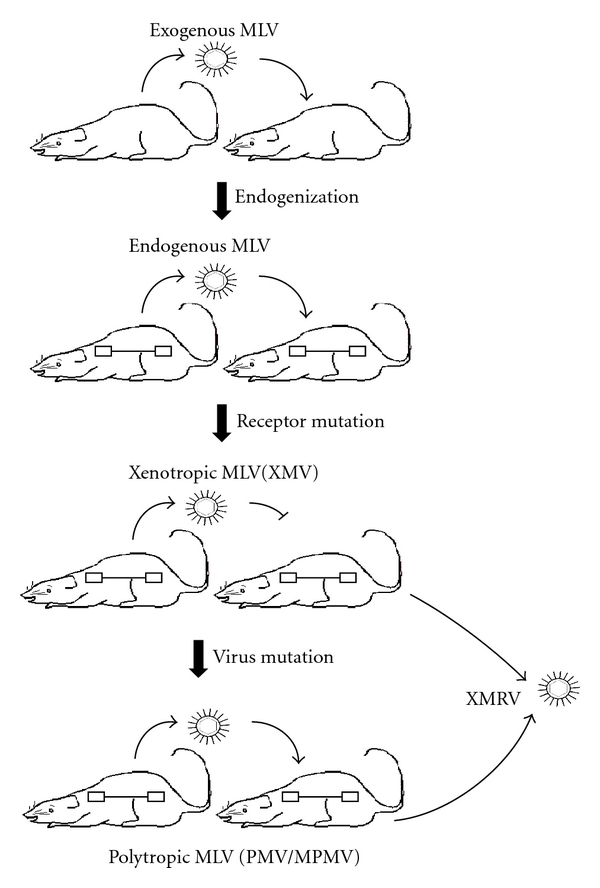
Schematic cartoon of the hypothetical origin of X-MLV, P-MLV, and XMRV. The mice illustrate the inferred pattern of MLV-host evolution, starting with infection of mice with an exogenous XMLV, its endogenization, selection for the resistant (Xpr1^n^) receptor allele, and the subsequent evolution of the polytropic (P-MLV and MP-MLV) viruses capable of using the mutant receptor.

As is the case with most host-pathogen arms races, evolution at the host-virus interface for MLVs and their mouse hosts is apparent on multiple levels. *Xmv* proviruses are considered to be the oldest MLV subgroup, a hypothesis supported by the genetic diversity among members, their failure to form a monophyletic clade, and their ancestral location in phylogenetic analyses (Figure 1) [10]. During the long course of their coexistence with endogenous and exogenous MLVs, many *Mus* subspecies have evolved ways to cope with such assaults. One example of a resistance mechanism is the evolution of variants of the Xpr1 receptor so that the modified allele no longer supports virus entry, conferring a selective advantage on the host that carries the new variant. Endogenous X-MLVs derived from proviruses in those mice could not infect the cells of their host organism. Viruses have in turn responded by incorporating mutations in their *env* genes, allowing them to use the new version of the receptor and giving rise to the evolution of polytropic and modified polytropic MLVs, which are also carried in numerous copies by many mouse genomes (Figure 2). Another level of resistance is encoded by a set of proteins induced as result of the expression of interferon (reviewed in [11]). One of the most important of these is the enzyme family APOBEC3 A-G (in humans) with one homolog in mice, Apobec3. APOBEC3's are incorporated into virions and cause high levels of dC to dU deamination on newly synthesized negative strand DNA, leading to substantial frequencies of G to A hypermutation in the positive strand (genome) of many retroviruses, effectively killing the provirus.

## 2. Origin of XMRV

Evidence for the presence of XMRV and other MLV-related viruses in human tissues have so far relied on various methods including fluorescence in situ hybridization (FISH), immunological detection of viral antigens and antibodies against them, isolation of infectious virus from human samples, and, finally, PCR amplification of MLV sequences [12–14]. We will discuss here only the last two issues, focusing on the relationship of XMRV and related sequences to endogenous MLVs and the likely events that gave rise to them. By the term “XMRV,” we refer only to the infectious viruses reported in the original papers [12, 13, 15]; related sequences detected by PCR amplification [14] will be referred to as “MLV-like” (Figure 1). We first focus on XMRV.

XMRV was originally described in a fraction of prostate cancer cases [12] and subsequently in a large fraction of patients with chronic fatigue syndrome (CFS) [13]. The association of XMRV with disease rapidly became controversial, however, when a large number of the studies in multiple patient cohorts, prompted by the initial reports, did not find XMRV, even though very sensitive detection methods were used (reviewed in [16]). The ensuing debate raised the issue of whether, on the one hand, the negative studies reflected poor technique or inappropriate study cohorts, or on the other, the positive results reflected contamination with a virus that did not, in fact, circulate in humans.

**Figure 3:**
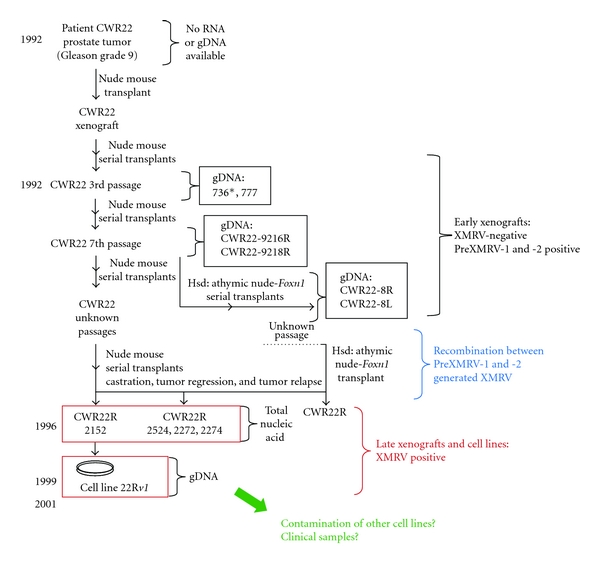
Derivation of the 22R*v1* cell line from prostate cancer xenografts in nude mice. Starting in 1992, the CWR22 prostate cancer was passaged repeatedly in nude mice until 1999, when the cell line was isolated. Samples of the tumor prior to 1996 were negative for XMRV, but contained small amounts of mouse DNA harboring both PreXMRV-1 and -2. Later samples were also positive for XMRV. Modified from Paprotka et al. [19].

In contrast to most other irreproducible reports of human “rumor” viruses dating back to the 1970s, XMRV was isolated as an infectious virus and grows to high titer on suitable human cell lines, from both diseases [13, 17]. An essentially identical virus was also found to be produced in large amounts by the 22R*v1* cell line [15], which had been derived from a human prostate cancer by repeated passage, over the course of 7 years, in the form of xenografts, in nude mice (Figure 3) [18]. This result was interpreted to imply that the tumor that eventually gave rise to the cell line was infected with XMRV at the outset. 

**Figure 4:**
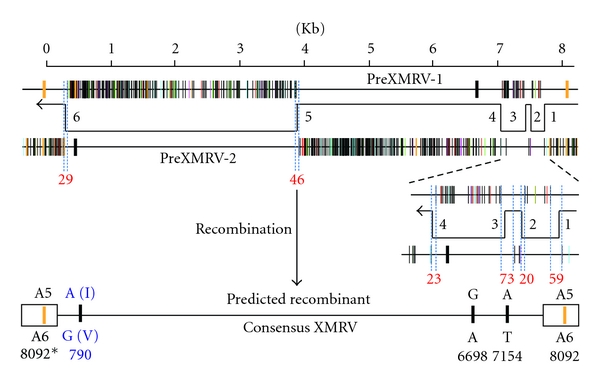
Recombination between PreXMRV-1 and -2 leads to XMRV. The sequences of the two proviruses identified by Paprotka et al. are shown schematically, with a vertical line indicating each position that differs from the XMRV consensus. The red line shows the positions of the 6 crossover events (common during retrovirus replication) that are inferred to have given rise to a virus differing from XMRV at only the 4 positions shown. Modified from Paprotka et al. [19].

XMRV shows high sequence similarity to endogenous *Xmv *proviruses [12], suggestive of a shared origin, but it is not identical to any of them. Despite considerable effort, we did not find it as a single endogenous provirus in any mouse genome [19]. However, the fortunate availability of DNA samples from various passages of the tumor xenografts from which 22R*v1* had been derived made it possible to trace the origin of XMRV [19]. Analysis of DNAs from early and late passages revealed that the virus was undetectable (less than about 1 provirus per 200 cells) in samples taken through 1993, showing that the original tumor did not contain XMRV (Figure 3). However, it was present in the xenografts performed after 1996, suggesting that the tumor had been infected by XMRV while being passaged through nude mice some time between 1993 and 1996. Moreover, two XMRV-related proviruses (PreXMRV-1 and -2) could be detected in the small amount of mouse DNA in the tumor samples, showing that the mouse strains used for the xenografts contained these two previously undescribed proviruses, whose genomes could recombine to generate a virus virtually identical to XMRV (Figure 4), providing the most likely explanation of how, where, and when the virus was created [19]. Retrovirus recombination is a very frequent event that occurs following infection of a cell with a virion containing two distinct genomes, produced by a cell containing the two parental proviruses. During the course of reverse transcription, the enzyme transfers repeatedly from one genome to the other, on average 4 times in the case of MLVs [20]. In the case of XMRV, a virion containing the PreXMRV-1 and PreXMRV-2 genomes and most likely containing proteins made by both proviruses and produced by a cell of the mouse host would have infected a human cell in the xenograft, leading to the infectious XMRV recombinant. The virus generated in this way could then spread through the tumor, perhaps conferring some selective advantage (such as increased growth rate or hormone independence) to the infected cells. Given the large number of stretches of identical sequence in the two parental proviruses, the probability of generating exactly the same recombinant more than once independently is negligible. These findings lead inexorably to the conclusion that the XMRV produced by 22R*v1* cells was created in the laboratory (or, more likely, the mouse room) and all subsequent isolates are likely to have originated from this single unique recombination event, almost certainly by cross-contamination in laboratories handling 22R*v1* cells and other susceptible cell lines.

As mentioned above, xenotropic MLVs typically cannot use the nonpermissive Xpr1^n^ receptor variant carried by most inbred strains [7]. When they encounter a permissive cell type, however, as happens when human cells are transplanted into mice, they can infect the cells of this newly encountered species. Acquisition of mouse endogenous retroviruses by heterologous cells occurs frequently, and many examples have been documented [9, 21–24]. Thus, the infection of the prostate cancer xenograft by an X-MLV in mice was far from unprecedented, but the details of the process, including the specific proviruses involved, had never before been worked out.

In the decade following its derivation in 1999 [18], the 22R*v1* cell line has been distributed worldwide and widely used for studies on prostate cancer biology. At the time of this publication, PubMed listed over 200 papers reporting its use from many different laboratories, none of which could have been aware before 2009 that it was producing 10^9^–10^10^ of virions per mL [15]. Given the ease with which viruses can spread from one culture to another even in virology laboratories that are aware of the problem, it is not hard to see how XMRV could have contaminated cultures in many different laboratories. Such contamination not only can give rise to false associations with human disease, but also, cause major changes in cellular processes, leading to alterations in cellular pathways under study. For these reasons, it is absolutely necessary, and should be routine practice, to continuously monitor cell lines for contaminating retroviruses.

**Figure 5:**
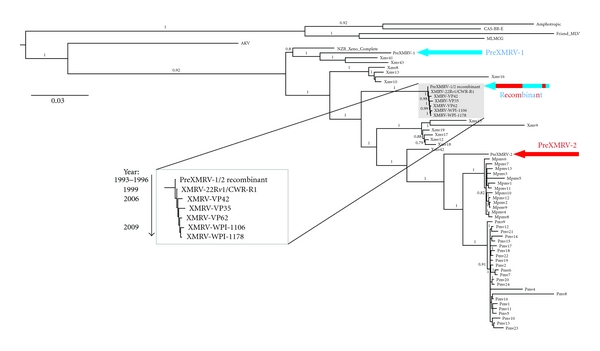
Phylogenetic analysis of MLVs and XMRV isolates. Note the positions of the two parents. The enlarged inset shows the XMRV portion of the tree, illustrating the ancestral position of the inferred recombinant and the subsequent possible progression of the virus. From Paprotka et al. [19].

Phylogenetic analysis of XMRV isolates in comparison with endogenous MLVs also strongly supports the same series of events. As can be seen in Figure 5, PreXMRV-1 groups with another *Xmv* subclade; PreXMRV-2 (which may itself be a recombinant) is closer, but not identical, to *Pmvs*. In the enlarged portion of the tree representing all published XMRV isolates, it can be seen that the inferred recombinant virus occupies a position ancestral to all XMRVs, followed by the virus produced by 22R*v1* cells, followed by the prostate cancer isolates, and followed finally by the CFS isolates, exactly consistent with the inferred sequence of infection and contamination events [19].

The two XMRV parents are not unique to nude mice: of 48 laboratory mouse strains tested, PreXMRV-1 is found in 6, PreXMRV-2 in 25 [Cingöz et al. In prep.], as well as in the NIH3T3 cell line, commonly used for many laboratory purposes, including preparation of MLV-based gene therapy vectors [25]. Three laboratory strains, including the two nude mice presumably used to passage the original tumor, contain both proviruses. They are also found in DNA from some wild-derived mice: PreXMRV-1 in *M. m. molossinus* and *M. m. castaneus*, and PreXMRV-2 in *M. m. domesticus*. Since the former mice are native to Asia, and the latter to Europe, it is improbable that they were ever together in the wild before human intervention.

In further support of the idea that XMRV has not infected humans is its high sensitivity to the primate APOBEC3G restriction factor, an interferon-inducible protein, which is constitutively expressed in some (but not all) cell types. Indeed, the extensive hypermutation caused by A3G makes it impossible to establish spreading infection of human PBMCs in cell culture [26] and, while proviral DNA persists in macaques experimentally infected with XMRV, this DNA is also heavily hypermutated [Kearney et al. In prep.]. Unlike PBMCs, a number of human cell lines, including the prostate cancer lines 22R*v1* and LNCaP do not express APOBEC3G. Combined with a favorable transcriptional environment [17], this property makes these cells particularly good hosts for XMRV infection, so it is not impossible that the virus could sometimes bypass the barrier to infect of blood cells.

## 3. MLV-Like Sequences

In an attempt to replicate the findings of XMRV in CFS patients, Lo et al. analyzed plasma and PBMCs from another cohort of CFS patients as compared to samples obtained at a different time and place from normal blood donors [14]. They reported that they were unable to detect XMRV using a specific PCR assay, but that, with somewhat less selective primers, they could detect fragments of sequence identical or closely related to some endogenous P-MLVs and MP-MLVs. Again, the sequences were detected much more frequently in samples from CFS patients than from the poorly matched controls. The close match of these sequences to endogenous MLVs present in over 100 copies per cell in laboratory and wild mice immediately raised the possibility of contamination of the samples with traces of mouse DNA. To counter this concern, Lo et al. also tested the samples for mouse mitochondrial DNA and, finding none, concluded that the results reflected infection of the patients, but not the controls, with polytropic-like MLV. In further support of this conclusion, they reported, that later samples from some of the same patients also contained MLV-like sequences. These sequences showed genetic differences from the earlier ones, leading them to conclude that there was ongoing virus replication and evolution.

**Figure 6:**
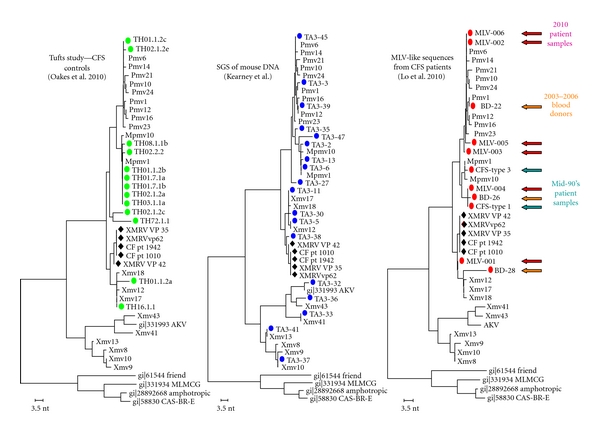
Phylogenetic analysis of MLV-like sequences. The three trees relate the endogenous MLV sequences described by Jern et al. [10] to the fragmentary MLV-like sequences found in (left, green dots) normal control DNAs by Oakes et al. [27], in (middle, blue dots) normal mouse DNA by M. Kearney (unpublished), and (right, red dots) in CFS samples (green arrows), normal blood donors (orange arrows), and samples drawn at a later date from the same CFS patients (red arrows) (data from Lo et al. [14]).

The possibility of a human gammaretrovirus circulating among the population and potentially having an association with human disease created considerable excitement in the field. A number of researchers proposed that XMRV- and MLV-related sequences represented related findings that strongly supported the conclusion of the association of MLV-like viruses with human disease. However, one should be very cautious about associating the two observations. Xenotropic and polytropic viruses are distinct MLV subclasses, a fact readily observed upon comparing the positions they occupy in phylogenetic trees [10] (Figures 1 and 5). XMRV is not found as a single endogenous provirus in any mouse strain tested [19], whereas the partial sequences detected by Lo et al. are very close or identical to proviruses found in mice [14]. XMRV sequences detected by Lombardi et al. are nearly identical to the XMRV sequences described by Urisman et al. and form a distinct clade among *Xmv* proviruses, while the MLV-like sequences of Lo et al. found in CFS and control samples are dispersed among other mouse endogenous proviruses [12–14] (Figure 6). The reported MLV-like sequences are bulk PCR fragments; no full length viral genome or infectious virus was recovered, as opposed to the Lombardi et al. study where infectious virus was recovered upon culturing patient samples. P-MLV fragments were detected following high numbers of PCR amplification cycles; no other detection methods were used and the results have not been replicated by any other study published to date. The differences between the two studies in the experimental methods used and the findings reported call for extreme caution to be taken before widely interpreting the data. Until we have a better understanding about the relationship between these viruses, the two studies should be treated separately and should not be taken as supporting or refuting each other.

Despite the inclusion of apparently adequate controls, mouse DNA contamination remains a significant concern. Extremely small amounts of DNA, from as little as one one-hundredth of a cell, contain enough provirus sequences to yield false positives with internal provirus-specific primers (Figure 6, middle panel). Two recent studies have described the detection of MLV-like sequences in samples from patients and/or healthy controls. In the first study by Oakes et al., positive amplification results were obtained from only 1 out of 111 samples tested from CFS patients, but from 18/36 healthy controls, which had been processed at a different time with a slightly different protocol [27]. In the second study, Robinson et al. found that 14/282 of UK prostate cancer cases, 5/139 of Korean, and 2/6 of Thai cases tested positive for amplification with XMRV primers [28]. As with the Lo et al. sequences, those of Oakes and Robinson fit perfectly within the endogenous MLV phylogeny (Figure 6, left panel). In both cases, some, but not all, MLV-positive samples were also positive for mouse mitochondrial DNA. To improve the sensitivity of detection of mouse DNA, we developed an assay that relies on PCR amplification of a small fragment from the LTR of intracisternal A particles (IAPs), LTR retroelements that are abundant (more than 1000 copies) in the mouse genome and not cross-reactive with any sequence in the human genome despite the presence of distantly related IAP elements [29]. Using this assay, both studies found that all samples that tested positive for MLV DNA sequences were also positive for IAP LTR sequences, implying sporadic mouse DNA contamination as the most likely source of the former sequences. The exact source of these mouse sequences has not fully pinned down. Trace amounts of mouse DNA could be already present in the laboratory reagents used or contamination could have occurred during sample collection or storage before they were even sent to the laboratories to be tested for XMRV. Four other studies further supported these findings, where potential sources of MLV genome contaminants, most likely from mouse genomic DNA, were discovered in commercially available laboratory reagents and kits, particularly Taq DNA polymerase containing a mouse monoclonal antibody [30–33]. It is possible that microtomes used for both laboratory and clinical samples could carry traces of mouse DNA over to human pathology samples, perhaps including the fixed and archived prostate cancer specimens analyzed by Robinson et al. [28]. Cross-contamination from a laboratory robot used for XMRV more than a year previously has also been reported [33]. Taken together, these findings call for caution before interpreting the data and the need for very sensitive assays to detect mouse DNA contamination, when endogenous MLV sequences are detected by PCR from human samples.

It is also important to emphasize that considerable caution should be exercised when attributing the origin of short PCR amplicons to a replicating virus, when the virus has itself not been isolated. Indeed, for reasons that are unclear, replication-competent P-MLVs have never been identified in mice, although their envelope genes can be donated to replication-competent recombinant viruses that arise and cause lymphoma in some strains of mice [35–37]. Until a real virus is identified, studies reporting detection of “virus” sequences must be taken as highly preliminary and suggestive, not definitive, evidence for human infection by P-MLVs.

A final problem with the sequences reported by Lo et al. is that they do not exhibit the sort of evolution expected for long-term infection. Although these authors reported that sequences that appeared to show evolutionary changes were obtained from some of the same patients ~15 years after the initial sampling [14], examination of the sequences made available in the GenBank database reveals that the “evolved” sequences are in fact very similar to other endogenous MLVs. (Figure 6, right panel) [27]. Rather than evolving as would be expected of a true infecting virus, these sequences therefore seem to be simply moving up and down the MLV phylogenetic tree. The conclusion that they represent MLV sequences amplified from trace mouse DNA contamination is inescapable.

## 4. Particular Issues regarding Detection of MLVs as Possible Human Pathogens

The initial reports of association between endogenous MLV-related viruses and human disease were attractive because of their biological plausibility: MLV-related viruses cause a similar variety of diseases in mouse models [1]; close contact between mice and humans can result in zoonotic infection; the virus isolated is highly infectious for at least some human tumor cell lines [17, 38]. As the studies presented here unfolded, however, a number of experimental issues regarding the possibility of detection of endogenous proviral sequences and their confusion with replicating viruses infecting human patients came to light. There are a number of lessons that should be heeded in the development of future studies.

First, low levels of mouse DNA contamination are very commonly found in laboratory reagents. In some cases, this DNA can be attributed to the inclusion of mouse-derived products, such as monoclonal antibodies in PCR reagents, or used for isolation of cells [27, 28, 30, 32, 33]; in others, the source of mouse DNA is less than clear, but given the close relationship of human and wild mouse activity, it is not hard to imagine that mice can leave traces in many places that can find their way into almost any laboratory reagent or supply. 

A second, related issue regards the provision of appropriate controls. Given the apparent sporadic nature of this sort of contamination, it is absolutely essential that controls and patient samples be exactly matched, not only for personal characteristics, such as age, gender, geography, and so forth, but also in the reagents and materials (tubes, needles, etc.) used to obtain and process the assay samples. Unfortunately, such caution is often not the case, particularly in retrospective studies [14]. Clinical samples are often collected as at least two separate groups, the simplest example being patient versus control samples. This lack of caution can result in detection of a contaminant in one set of samples and not the other, resulting in false association of virus with human disease. Differential association between two different groups of clinical specimens can occur, even if the detected entity is an artifact. In fact, in the study by Oakes et al. [27], MLV sequences were detected preferentially in healthy controls drawn at a later time and processed by a slightly different method. One possible explanation for such erroneous associations is that the two groups might have been handled differently, collected at different times by different people at different locations using different reagents, supplies, or methods. Furthermore, tubes containing patient samples may have been accessed more frequently and under different circumstances than controls. For these reasons, blinding of investigators to which samples are cases and which are controls for such studies is crucial. Examples of such false associations have persisted, even when independent laboratories had confirmed findings after the initial report [39]. 

Third, even in the absence of contaminating mouse DNA, a different complication arises from retrovirus-contaminated cell lines used in the laboratory. There are multiple documented cases of such contamination events, which appear to be quite common among laboratories working with retroviruses [8, 15, 40–43]. Even in laboratories that do not work with retroviruses, there are examples of cell lines producing replication-competent retroviruses. Contamination of cell lines with retroviruses could occur through cross-contamination of previously uninfected cells with a virus grown or handled on other cells nearby. As long as the virus is replication-competent and can establish an efficient infection, it could eventually take over the entire culture even with trace amounts of starting virus. 

The overall lesson to be learned here is that extreme measures are required to avoid false associations of mouse viruses with disease, including: (1) rigorous use of highly sensitive assays for detecting mouse DNA contamination of supplies and reagents; (2) frequent testing of cell cultures used in the laboratory for undetected infection with an MLV or another virus; (3) the use of controls that are exactly contemporaneous to the cases and obtained by precisely the same methods using the same materials and reagents. As a few recent papers indicate [30, 32, 33], these conditions are not easy to achieve, but only laboratories that do so can make credible claims to the discovery of new human infections.
